# Definition of mortality-associated thermal markers for an extreme heat alert system in Belo Horizonte, Minas Gerais, Brazil

**DOI:** 10.1590/1980-549720260048

**Published:** 2026-08-28

**Authors:** Taíza Pinho Barroso Lucas, Amanda Silva Magalhães, Daniele Gomes Ferreira, Gabriel Andrade Camilo, Magda do Carmo Parajára, Fernanda Carvalho de Menezes, Aline Dayrell Ferreira Sales, Waleska Teixeira Caiaffa

**Affiliations:** I Prefeitura de Belo Horizonte, Special Coordination for Climate Change – Belo Horizonte (MG), Brazil.; II Universidade Federal de Minas Gerais, Observatory for Urban Health in Belo Horizonte – Belo Horizonte (MG), Brazil.; III Universidade Federal de Minas Gerais, School of Medicine, Graduate Program in Public Health – Belo Horizonte (MG), Brazil.; IV Universidade Federal de Minas Gerais, Institute of Geosciences, Graduate Program in Geography – Belo Horizonte (MG), Brazil.

**Keywords:** Heat waves, Ambient temperature, Time series studies, Public health surveillance, Urban health

## Abstract

**Objective:**

To estimate daily maximum temperature markers for defining heat wave alert levels in Belo Horizonte, Minas Gerais, Brazil.

**Methods:**

the association between daily maximum temperature and mortality was estimated using quasi-Poisson regression with distributed lag non-linear models, considering lags from 0 to 21 days and adjustment for temporal trend, seasonality, and day of the week. The minimum mortality temperature was used as the reference for estimating relative risks, and high percentiles supported the definition of operational thresholds.

**Results:**

The minimum mortality temperature was 29.3°C. Mortality risk increased at higher percentiles, with relative risks of 1.04 at the 90th percentile; 1.09 at the 95th percentile; 1.17 at the 98th percentile; and 1.26 at the 99th percentile. Four levels based on maximum temperature and thermal persistence were proposed: Alert 1, mild heat wave (32.0–33.1°C/3 days); Alert 2, moderate heat wave (33.2–34.6°C/3 days); Alert 3, intense heat wave (34.7–35.8°C/3 days); and Alert 4, extreme heat wave (>35.8°C/2 days). A higher frequency of heat waves was observed in years associated with El Niño.

**Conclusion:**

Markers based on high percentiles of maximum temperature produced levels consistent with the increased mortality risk, with potential application in public health surveillance and response.

## INTRODUCTION

Heatwaves have increased in both frequency and intensity in the context of climate change, emerging as one of the climate extremes with the greatest impact on public health^1,2^. In urban areas of Brazil, their occurrence extends beyond the meteorological dimension, resulting in disproportionate impacts on populations affected by inequalities in housing conditions, urban infrastructure, and access to health services^3,4^.

Historical events in several countries have demonstrated a substantial increase in morbidity and mortality associated with prolonged exposure to extreme heat, including the 1995 Chicago heatwave^5,6^ and the 2003 European heatwave, both of which resulted in tens of thousands of excess deaths^7^. Epidemiological studies and systematic reviews have consistently reported an association between high temperatures and increased mortality, particularly from cardiovascular and respiratory causes, as well as a higher incidence of adverse health outcomes, including renal impairment, dehydration, fluid and electrolyte imbalances, hospital admissions, and emergency department visits^8–12^. These effects are generally more pronounced among older adults, children, heat-exposed workers, and socially vulnerable populations^13–16^.

There is no universally accepted definition of a "heatwave," as its characterization depends on local climatological conditions and the associated health impacts. International guidelines and public health research recommend risk-based approaches that use temperature thresholds relative to the local historical distribution^2,17^. In Brazil, the operational criterion adopted by the National Institute of Meteorology (*Instituto Nacional de Meteorologia* – Inmet) defines a heatwave as a period of five or more consecutive days during which daily maximum temperatures exceed the local climatological average by at least 5°C^18^. Although this definition is widely used, it may have limitations for epidemiological applications. In contrast, approaches based on temperature percentiles have been shown to be more suitable for capturing local climatic characteristics, identifying heatwaves across diverse settings, and evaluating their health impacts^19–22^.

Persistent heat, low relative humidity, air pollution, and other adverse environmental conditions may exacerbate health risks during extreme heat events^2,22^. Accordingly, studies have emphasized that the definition of heatwaves for epidemiological and operational purposes should account for local climatic characteristics and their relationship with health outcomes, as no universal thermal threshold is applicable across different regions^23^. Approaches based on high-temperature percentiles and relative risk indicators have been widely adopted in the development of early warning systems and public health response protocols^17,24,25^.

In Brazil, Bitencourt et al. reported increases in the frequency, duration, and intensity of heatwaves over recent decades, associated with both climate variability and regional warming^21^. Belo Horizonte (BH), one of the country's major state capitals, exhibits characteristics that pose challenges for environmental management, including high urban density, extensive verticalization in central areas, and an uneven distribution of vegetation cover^26^. The city has experienced an increase in extreme heat events, particularly during spring, the transitional season preceding the rainy period characteristic of the Tropical Continental climate^27^. In addition, climate variability associated with the El Niño–Southern Oscillation (ENSO) may influence temperature and precipitation extremes in Brazil, intensifying temperature anomalies during its warm phase^28^.

Several countries have implemented heat warning systems tailored to their climatic conditions and designed to mitigate adverse health impacts^17,24^. In the United Kingdom, the warning system has evolved to place greater emphasis on health-related outcomes^29^, while Hong Kong developed the Hong Kong Heat Index^30^. In Brazil, the city of Rio de Janeiro adopted an index that combines air temperature and relative humidity to support the implementation of preventive measures^31^.

In BH, refinement of the Municipal Plan for Climate Risk and Disaster Management requires epidemiologically informed parameters to support intersectoral responses to extreme heat events. This study aimed to establish daily maximum temperature thresholds for defining alert levels and characterizing heatwaves in BH, thereby providing a theoretical and methodological framework for climate risk management in the municipality.

## METHODS

### Study area

BH is located in Southeastern Brazil, encompasses an area of 331.4 km^2^, and has a population of approximately 2.3 million inhabitants^32^. Its climate is classified as Tropical Continental Highland, within the Montane Tropical Subdomain and the Central Brazil Tropical Domain, according to the updated classification of Brazilian climatology proposed by Novais^27^.

The physical characteristics of BH differ between its southwestern-southern and northern sectors. The southern sector is characterized by geological formations of the Minas Supergroup, composed of weathering-resistant metasedimentary rocks, and is associated with undulating, dissected terrain and elevations exceeding 1,300 m^33^. These geomorphological features influence urban thermal dynamics, particularly in areas adjacent to the Serra do Curral, which exhibit lower average temperatures and higher precipitation levels than other parts of the municipality^34^. The combined effects of topography, elevation, thermal gradients, and low relative humidity, associated with the influence of the South Atlantic Subtropical Anticyclone (SASA), together with extensive soil sealing, contribute to heterogeneous patterns of heat exposure across the municipal territory^35^.

### Data sources

Mortality data were obtained from the Mortality Information System (*Sistema de Informação sobre Mortalidade* – SIM). Daily records of all-cause deaths among residents of BH were extracted, covering the years 2014–2024. The choice to focus on all-cause mortality aims to capture the systemic impact of heat exposure on municipal public health.

Meteorological data were obtained from the monitoring network of Inmet. The municipality is served by one conventional station and two automatic stations located in areas with distinct geoecological characteristics. The Pampulha Automatic Station (*Estação Automática da Pampulha* – EAP; code A521; lat: −19.88; long: −43.97), situated in the northwestern sector of the city, was selected because of its spatial and altimetric representativeness. Located at an elevation of 854 m, the station closely reflects the average elevation of the BH urban area. Despite the city's complex urban morphology and the presence of microclimatic variability, EAP occupies a strategic position relative to the centroid of the urban footprint, providing a representative characterization of the municipality's climatic conditions. Daily records of maximum (Tmax) and minimum (Tmin) temperatures, along with their respective monthly averages, were analyzed for the 2014–2024 period. Thermal anomalies were calculated using the 1991–2020 Climatological Normals as the reference baseline^36^.

### Statistical analyses

#### Step 1

An exploratory analysis was conducted to characterize the behavior of Tmax and Tmin during the 2014–2024 period in comparison with the 1991–2020 climatological normals^36^. The 25^th^ and 75^th^ percentiles of monthly Tmax and Tmin were also calculated to describe the thermal variability observed throughout the study period. Heatwave episodes were identified based on a thermal anomaly of 5°C above the reference climatological mean, in accordance with the criterion adopted by Inmet^18^. For the purposes of this study, a minimum duration of three consecutive days was established as the temporal persistence threshold.

The use of high percentiles of the Tmax distribution complemented Inmet's climatological approach by incorporating local specificities and enabling an assessment of their association with the relative risk of mortality in the municipality. Studies have demonstrated that approaches based on temperature percentiles are more suitable for epidemiological analyses and for defining local risk thresholds^19–23^.

This calibration is based on guidelines from the World Health Organization (WHO) and the World Meteorological Organization (WMO), which recommend adapting heatwave definition criteria to local climatic characteristics and associated health impacts^17^. It should be noted that the three-consecutive-day threshold has already been institutionally adopted by the Municipal Civil Defense for risk management in BH, thereby providing operational applicability to the proposed markers.

Following the identification of heatwave episodes, the influence of interannual climate variability, specifically the El Niño and La Niña phases, was examined^28,37^. The classification of ENSO events was based on the Oceanic Niño Index (ONI), calculated from quarterly sea surface temperature anomalies in the Niño 3.4 region of the Equatorial Pacific, according to the criteria established by the National Oceanic and Atmospheric Administration (NOAA)^37^. Events classified as very strong corresponded to ONI values ≥2.0°C.

For the subsequent modeling, Tmax was selected due to its widespread use in the characterization of heat extremes^19–22^ and its association with increased mortality, as documented in epidemiological studies^8–12^. This approach enabled the preservation of the daily temporal variability required to estimate the effects associated with extreme heat.

#### Step 2 – Identification of persistent heat markers

To determine the thermal indicator most representative of extreme events, correlation and linear regression analyses were performed based on the episodes identified in Stage 1. Pearson's correlation analysis was initially applied between Tmax and dry-bulb temperatures recorded at 06:00, 10:00, 14:00, and 18:00, representing dawn, daytime warming, the afternoon thermal peak, and the onset of nocturnal cooling, respectively. Subsequently, simple and multiple linear regression models were fitted to assess the explanatory capacity of hourly combinations from the diurnal cycle in predicting Tmax. The complete set of hourly correlations is presented in Table S1 (supplementary material).

#### Step 3 – Intra-day and inter-day temperature variation

Variations in Tmax throughout the diurnal cycle and across days were investigated using a Generalized Additive Model (GAM)^38^ for descriptive purposes. Tmax was defined as the response variable, whereas time of day and temporal sequence were included as covariates. The model incorporated cyclic splines for the time-of-day variable and penalized cubic splines for the temporal dimension, allowing adjustment for seasonal and temporal trends. This approach enabled the modeling of intra- and inter-day variations while avoiding overfitting and characterizing thermal persistence during heatwave episodes^38^.

#### Step 4 – Modeling the association between temperature and mortality

A distributed lag non-linear model (DLNM) with a quasi-Poisson distribution^39,40^ was used to estimate the association between air temperature and all-cause mortality. For the exposure-response relationship, a natural cubic spline with internal knots at the 50^th^ and 90^th^ percentiles of the temperature distribution was applied^39,40^. For the lag-response relationship, a natural cubic spline with three internal knots equally spaced on the logarithmic scale was used, considering a lag period from 0 to 21 days^39,41^. Adjustment for seasonality and long-term trends was performed using a time-stratification variable including year, month, and day of the week. The final model specifications were selected based on the lowest Quasi-Akaike Information Criterion (Q-AIC) value, as detailed in Table S2 (supplementary material).

Prior to selecting Tmax as the primary predictor, the applicability of the Heat Adjusted Air Temperature (HAAT) index was evaluated. This assessment involved analyzing correlations between daily Tmax, dry-bulb temperatures recorded at 06:00, 10:00, 14:00, and 18:00 during the episodes identified in Stage 1, and the corresponding relative humidity values. However, the analysis demonstrated that the conversion of the NOAA minimum threshold (80°F, approximately 26.7°C) and the relative humidity requirement of ≥40% rendered the index unsuitable for the climatic conditions of BH, which are characterized by low humidity, particularly during heatwave events (Figure S1 – supplementary material)^42^. This limitation differs from the conditions observed in heat–mortality studies conducted in Rio de Janeiro^43^, where maritime influence has a greater role in local thermal conditions.

#### Step 5 – Risk thresholds and parameters for the Heat Protocol

Based on the DLNM model results, the Minimum Mortality Temperature (MMT), defined as the temperature associated with the lowest mortality risk, was estimated. To avoid unstable estimates at the distribution extremes, MMT was restricted to the 1^st^ to 99^th^ percentiles of daily Tmax^44^. Relative Risks (RR) and 95% confidence intervals (95%CI) were estimated using the MMT as the reference temperature.

To assess the robustness of the findings, sensitivity analyses were conducted by testing different knot positions along the exposure-response dimension and varying lag periods, with the model providing the best overall fit based on Q-AIC being prioritized^45^. Based on the estimated RRs, MMT, and the persistence of consecutive days of extreme heat, the thermal parameters underlying the municipality's Heat Protocol alert levels were defined. Analyses were performed using R software (version 4.4.2) with the dlnm^39^ and gnm^46^ packages, while supplementary descriptive processing was conducted using spreadsheets.

##### Data availability statement:

The entire dataset supporting the results of this study is available from the corresponding author upon request. The dataset is not publicly available due to the integration of databases performed by the authors.

## RESULTS

### Step 1

Meteorological data from the 2014–2024 period revealed systematic deviations from the 1991–2020 climatological normals. The mean Tmax during the period was 28.4°C, approximately 1°C above the historical average, whereas the mean minimum temperature (17.6°C) remained slightly below the climatological average.

Monthly Tmax anomalies revealed a predominance of positive deviations throughout the analyzed period (Figure S2A – supplementary material), ranging from +0.2°C to +2.3°C. This warming trend was most pronounced during the September–November quarter, reaching its peak in September (approximately +2.3°C), which indicates an intensification and prolongation of heat during the transition to the rainy season. This pattern was confirmed by the absolute temperature values (Figure S2B), in which mean maximum temperatures consistently exceeded 30°C during this period, highlighting the high seasonal exposure of the BH population to heat.

In contrast, Tmin exhibited persistent negative anomalies between March and August (Figure S2A), with the lowest mean minimum temperatures occurring in July (-1.3 to −1.5°C), indicating greater nighttime cooling during the dry season. From September onward, a shift in the thermal pattern was observed, with a gradual increase in Tmin through December, approaching or exceeding the climatological normals in most years, except for 2021 and 2022. This pattern highlights asymmetric warming driven by increasing maximum temperatures, particularly during the winter-to-spring transition (Figure S2B). Such asymmetry characterizes urban thermal exposure by demonstrating the greater contribution of daytime maximum temperatures to extreme heat events.

Annual heatwaves were identified based on daily Tmax records (Table 1). The year 2015, influenced by a very strong El Niño event according to the ONI index, recorded the highest number of events in the series, concentrated between September and November. The years 2019 and 2024 also exhibited a high frequency of heatwaves, associated with the influence of warm ENSO phases. In contrast, years influenced by La Niña showed lower thermal persistence and a lower frequency of episodes classified at the highest alert levels. Although 2023 had only three events, it included the heatwave with the longest duration and greatest intensity during the analyzed period. During this event, the highest absolute temperature in the series (38.4°C) was recorded, exceeding the monthly climatological normal by more than 10°C and occurring alongside concurrent increases in minimum temperatures.

**Table 1:** Heatwaves in Belo Horizonte (MG), from 2014 to 2024.

Year	Start date	End date	Number of days
2014	09/29/2014	10/01/2014	3
	10/12/2014	10/15/2014	4
	10/17/2014	10/19/2014	3*
2015	09/22/2015	09/27/2015	6*
	10/14/2015	10/17/2015	4*
	10/20/2015	10/22/2015	3*
	11/03/2015	11/07/2015	5*
	11/10/2015	11/13/2015	4*
2016	10/19/2016	10/21/2016	3†
2017	10/20/2017	10/22/2017	3†
2018	-	-	-
2019	09/10/2019	09/13/2019	4
	09/17/2019	09/21/2019	5
	11/04/2019	11/06/2019	3
	11/08/2019	11/11/2019	4
2020	09/18/2020	09/20/2020	3†
	09/27/2020	10/03/2020	7†
	10/06/2020	10/09/2020	4†
2021	09/09/2021	09/11/2021	3†
	09/13/2021	09/15/2021	3†
2022	-	-	-
2023	07/11/2023	07/13/2023	3*
	09/23/2023	09/28/2023	6*
	11/09/2023	11/19/2023	11*
2024	05/01/2024	05/03/2024	3*
	05/12/2024	05/14/2024	3*
	08/23/2024	08/25/2024	3
	09/02/2024	09/05/2024	4

* El Niño event;

† La Niña event.

Source: Brazilian National Institute of Meteorology and the National Oceanic and Atmospheric Administration.

### Step 2

Table S3 (Supplementary Material) presents the regression models relating hourly temperatures to Tmax during heatwave episodes (2014–2024). The 6:00 temperature (T06h) showed a moderate correlation with Tmax (R = 0.46; R² = 0.21), with performance similar to that of the 10:00 temperature (R = 0.44; R² = 0.20). In contrast, the 14:00 temperature (T14h) demonstrated a strong association with Tmax (R = 0.80; R² = 0.64) and exhibited the lowest individual standard error (0.81°C). The multiple model (M4), which integrated readings from 10:00, 14:00, and 18:00, achieved the best fit (adjusted R² = 0.76), indicating that thermal persistence throughout the diurnal cycle is a key determinant of heatwave magnitude in the municipality.

### Step 3

GAM modeling confirmed a consistent diurnal pattern during extreme heat episodes. The observed behavior corroborates the findings from Stage 2, identifying the 14:00 temperature as a key indicator of Tmax in BH. A progressive increase in temperature was observed throughout the morning, reaching a peak plateau between 14:00 and 16:00, followed by a gradual decline during nighttime hours. The temporal spline fit revealed thermal persistence across consecutive days, reinforcing the prolonged nature of heat exposure during extreme heat episodes.

### Step 4

**Figure 1:**
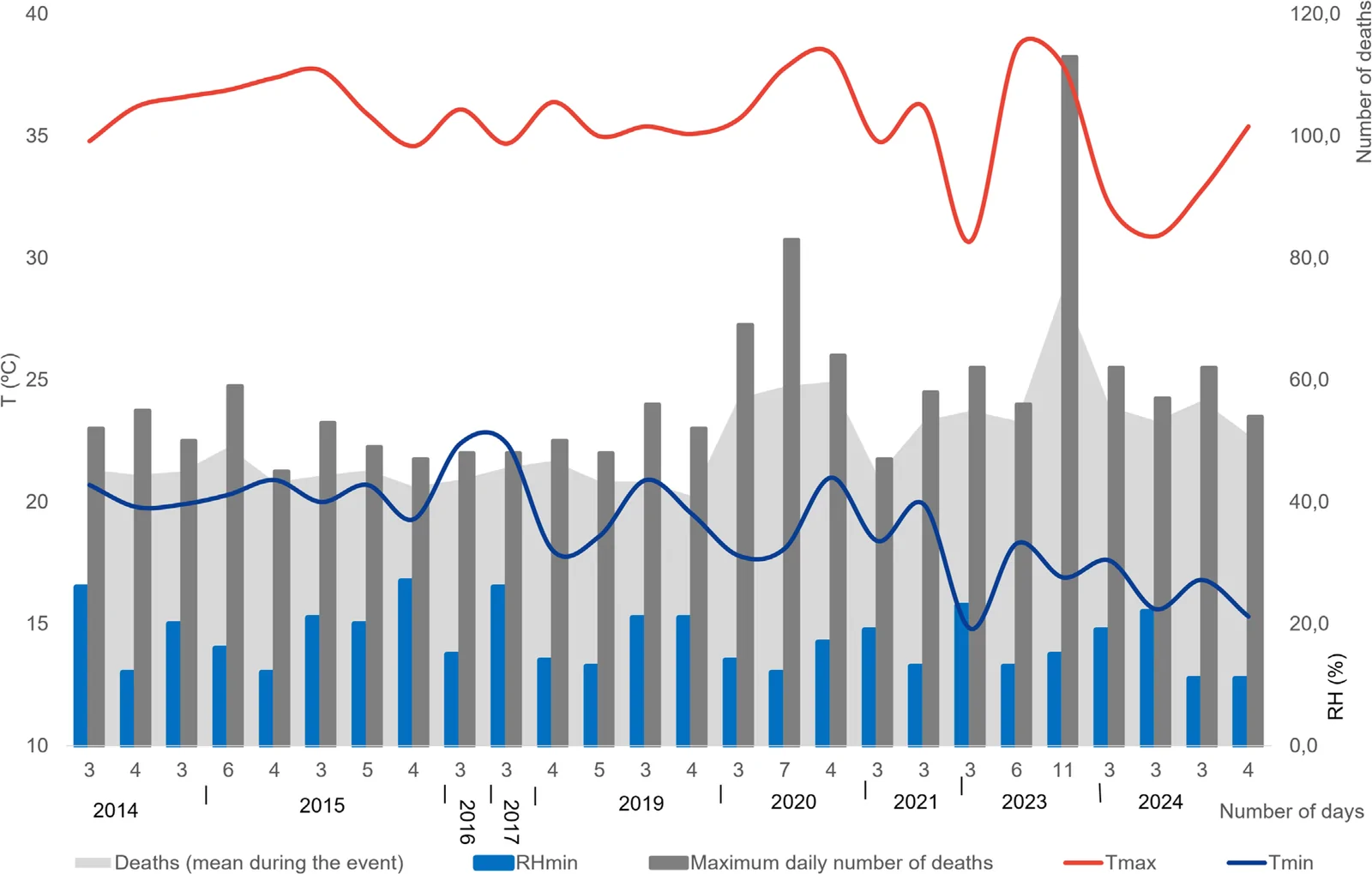
Heat wave events in Belo Horizonte (MG) (2014-2024) and their relationship with mortality and meteorological variables.

During the analyzed period, the daily average number of deaths from all causes was 47.6. The increase in mortality observed in 2020 was associated with the pandemic context and showed no correlation with temperature extremes. In contrast, heatwave episodes in September 2015 and, most notably, November 2023 exhibited a consistent pattern of increased mortality coinciding with peaks in Tmax and Tmin and a marked decrease in RH (Figure 1). During the heatwaves, all RH values remained below 40%.

Columns: maximum daily number of deaths per event; shaded area: mean number of deaths during the event; lines: maximum temperature (Tmax) and minimum temperature (Tmin); blue bars: minimum relative humidity; lower axis: duration (number of days).

Source: Brazilian National Institute of Meteorology and the Mortality Information System (Sistema de Informação sobre Mortalidade/DataSUS).

Stratified analysis revealed that approximately 40% of the evaluated days exhibited temperatures above the MMT (29.3°C). A progressive dose-response effect was observed: the average number of deaths increased from 48.0/day (on days above the MMT) to 55.1/day on days with Tmax ≥35.8°C (P99), as shown in Table 2.

**Table 2:** Number of days and mean daily mortality by temperature range (2014–2024) in Belo Horizonte (MG), Brazil.

Tmax (°C)	Number of days	Mean deaths per day
≥29.3°C (MMT; P59)	1,626	48.0
≥32.0°C (P90)	423	48.7
≥33.1°C (P95)	197	49.8
≥34.6°C (P98)	80	51.4
≥35.8°C (P99)	40	55.1

Source: Brazilian National Institute of Meteorology and the Mortality Information System (*Sistema de Informação sobre Mortalidade*/DataSUS).

**Figure 2:**
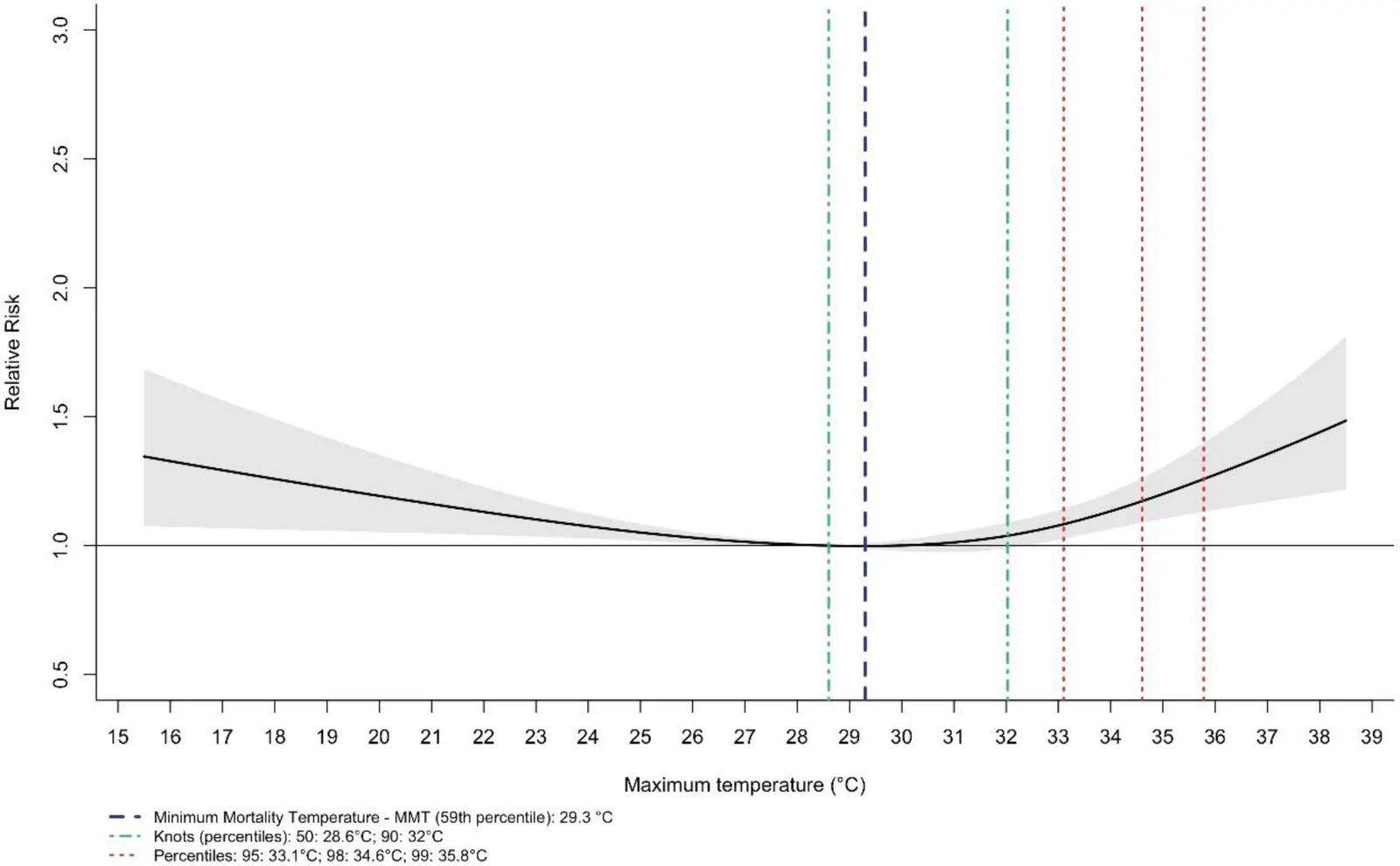
Cumulative association between maximum temperature (Tmax) and all-cause mortality in Belo Horizonte (MG) (lag 0-21 days), using the Minimum Mortality Temperature as the reference.

DLNM modeling confirmed the non-linear relationship and the "U-shaped" risk curve (Figure 2). Above the MMT (P59), the mortality RR showed a marked increase at higher percentiles, reaching RR = 1.09 (95%CI 1.04–1.14) at P95 and RR = 1.26 (95%CI 1.13–1.41) at P99, considering the cumulative effect over a lag period of 0 to 21 days.

The solid line represents the exposure-response curve, and the shaded area represents the 95% confidence interval. The blue dashed line indicates the Minimum Mortality Temperature (59^th^ percentile); the green dotted lines indicate the model knots (50^th^ and 90^th^ percentiles); and the red dash-dot lines indicate the extreme percentiles (95^th^, 98^th^, and 99^th^).

Source: Brazilian National Institute of Meteorology and the Mortality Information System (*Sistema de Informação sobre Mortalidade*/DataSUS).

### Step 5

Following the DLNM modeling, alert levels were defined for the BH Protocol (Table 3), adopting Tmax as the reference variable. The thermal thresholds were based on the MMT (29.3°C) and the 90^th^, 95^th^, 98^th^, and 99^th^ percentiles, which demonstrated a progressive increase in the relative risk of mortality.

**Table 3:** Heat alert levels for Belo Horizonte (MG), Brazil.

Level/Alert	Criteria for alert level (Tmax and number of consecutive days)	Classification	Health risk
Initial	29.3°C<Tmax≤31.9°C	ADVISORY	Very low risk
Alert 1	32.0°C≤Tmax≤33.1°C 3 consecutive days	MILD HEAT WAVE	Low risk
Alert 2	33.2°C≤Tmax≤34.6°C 3 consecutive days	MODERATE HEAT WAVE	Moderate risk
Alert 3	34.7°C≤Tmax≤35.8°C 3 consecutive days	SEVERE HEAT WAVE	High risk
Alert 4	Tmax>35.8°C 2 consecutive days	EXTREME HEAT WAVE	Very high risk

Source: Brazilian National Institute of Meteorology and the Mortality Information System (*Sistema de Informação sobre Mortalidade*/DataSUS).

The initial level (Attention) comprises the temperature range immediately above the MMT (29.3–31.9°C). For Alert levels 1, 2, and 3 (mild, moderate, and intense heatwaves), the criterion established was the occurrence of three consecutive days with temperatures within the ranges of 32.0–33.1°C, 33.2–34.6°C, and 34.7–35.8°C, respectively. For Alert level 4 (extreme), a threshold of two consecutive days above 35.8°C (P99) was adopted, given the critical risk observed at this percentile.

The hourly distribution confirmed a well-defined diurnal pattern, with thermal peaks concentrated between 12:00 and 17:00 and the maximum values occurring between 14:00 and 15:00 (Figure S3 – supplementary material). This pattern was particularly evident at Alert Level 4, reinforcing Tmax as the most robust predictive marker for operationalizing the alert system and defining periods of heightened thermal vulnerability in BH.

## DISCUSSION

The results suggest an association between increasing Tmax, the persistence of consecutive days of extreme heat, and increased mortality in BH, without implying uniformity across the analyzed episodes. This pattern highlights the limitations of heatwave definitions based solely on meteorological criteria when applied to public health contexts and supports the adoption of impact-based criteria calibrated to local climatic characteristics, as recommended by the WHO and WMO^17,23^.

The non-linear relationship between temperature and mortality, characterized by a "U-shaped" curve and an MMT of 29.3°C, is consistent with evidence from multicenter studies on temperature and mortality^40,44^. In BH, the increase in risk was more pronounced at temperatures above the MMT, particularly at the upper percentiles of Tmax. The progressive increase in relative risk beginning at P90, reaching an RR of 1.26 at P99 (>35.8°C), provides an empirical basis for differentiating alert levels and incorporating thermal persistence as an operational criterion^22,23,45^.

The inapplicability of the HAAT index in BH stems from high local climatic variability and the persistence of low RH during extreme heat events. This condition differs from that observed in Rio de Janeiro, where maritime influence plays a greater role in thermal parameters, favoring the use of an index that combines temperature and RH^31,43^. In BH, extreme heat events are frequently associated with atmospheric stability, subsidence, reduced cloud cover, and low RH in Southeastern Brazil^47^. This pattern reinforces the use of Tmax as the indicator for the local warning system.

As a city with a Tropical Continental Highland climate, BH exhibits marked seasonality, with heat peaks occurring in spring, a transitional period preceding the rainy season^27^. Hourly analysis presented in Figure S3 (supplementary material) reinforces this pattern by showing that maximum temperatures are concentrated between 12:00 and 17:00, a period of increased population exposure due to work activities and urban commuting. The higher frequency of heatwaves during El Niño years is consistent with evidence regarding the influence of warm ENSO phases on temperature and precipitation extremes in Brazil^28^. The severe 2014–2015 drought in Southeastern Brazil and the exceptional warming observed in 2023 highlight the importance of interannual climate variability in intensifying temperature extremes in Brazil, a factor that should be considered when improving public health warning and response systems^17,47–49^.

The results are consistent with national and international experiences regarding heat-health warning systems, including that of Rio de Janeiro, which also employs graduated risk levels^17,24,29,31^. The proposal for BH differs by anchoring thresholds to Tmax and thermal persistence, thereby reflecting local characteristics related to continentality, altitude, and seasonality. This approach facilitates institutional communication and decision-making amid climate variability, including El Niño episodes, and supports weekly forecasting. A recognized limitation is the reliance on a single weather station, which restricts the assessment of intra-urban thermal heterogeneity resulting from topography, vegetation cover, and land use^26,33,34^. Future research incorporating microclimatic data and analyses stratified by vulnerable groups may further refine the proposed thresholds spatially. Nevertheless, the established markers provide an operational benchmark for public health surveillance and response, contributing to a more proactive institutional approach to extreme heat events.
