# Supplementary material for: Definition of mortality-associated thermal markers for an extreme heat alert system in Belo Horizonte, Minas Gerais, Brazil
Source: Rev Bras Epidemiol. 2026 Aug 28;29:e260048. doi: 10.1590/1980-549720260048 (PMC13524307; doi:10.1590/1980-549720260048)
Supplement: Supplementary file 1 [file 1980-5497-rbepid-29-e260048-suppl1.docx]

**Material Suplementar**

**Tabela S1: Modelo de regressão linear geral entre todas as temperaturas horárias e a Tmax durante episódios de ondas de calor em Belo Horizonte de 2014-2024.**

|  | 0h | 1h | 2h | 3h | 4h | 5h | 6h | 7h | 8h | 9h | 10h | 11h | 12h | 13h | 14h | 15h | 16h | 17h | 18h | 19h | 20h | 21h | 22h | 23h |
| --- | --- | --- | --- | --- | --- | --- | --- | --- | --- | --- | --- | --- | --- | --- | --- | --- | --- | --- | --- | --- | --- | --- | --- | --- |
| 0h | 1 |  |  |  |  |  |  |  |  |  |  |  |  |  |  |  |  |  |  |  |  |  |  |  |
| 1h | 0,98 | 1,00 |  |  |  |  |  |  |  |  |  |  |  |  |  |  |  |  |  |  |  |  |  |  |
| 2h | 0,96 | 0,99 | 1,00 |  |  |  |  |  |  |  |  |  |  |  |  |  |  |  |  |  |  |  |  |  |
| 3h | 0,94 | 0,95 | 0,97 | 1,00 |  |  |  |  |  |  |  |  |  |  |  |  |  |  |  |  |  |  |  |  |
| 4h | 0,92 | 0,93 | 0,93 | 0,98 | 1,00 |  |  |  |  |  |  |  |  |  |  |  |  |  |  |  |  |  |  |  |
| 5h | 0,90 | 0,91 | 0,92 | 0,95 | 0,97 | 1,00 |  |  |  |  |  |  |  |  |  |  |  |  |  |  |  |  |  |  |
| 6h | 0,88 | 0,88 | 0,90 | 0,94 | 0,94 | 0,97 | 1,00 |  |  |  |  |  |  |  |  |  |  |  |  |  |  |  |  |  |
| 7h | 0,86 | 0,85 | 0,87 | 0,87 | 0,87 | 0,87 | 0,90 | 1,00 |  |  |  |  |  |  |  |  |  |  |  |  |  |  |  |  |
| 8h | 0,84 | 0,83 | 0,84 | 0,79 | 0,79 | 0,79 | 0,80 | 0,94 | 1,00 |  |  |  |  |  |  |  |  |  |  |  |  |  |  |  |
| 9h | 0,84 | 0,82 | 0,83 | 0,78 | 0,76 | 0,76 | 0,76 | 0,88 | 0,97 | 1,00 |  |  |  |  |  |  |  |  |  |  |  |  |  |  |
| 10h | 0,83 | 0,81 | 0,80 | 0,76 | 0,75 | 0,75 | 0,73 | 0,83 | 0,91 | 0,96 | 1,00 |  |  |  |  |  |  |  |  |  |  |  |  |  |
| 11h | 0,78 | 0,76 | 0,75 | 0,69 | 0,68 | 0,70 | 0,67 | 0,78 | 0,88 | 0,91 | 0,96 | 1,00 |  |  |  |  |  |  |  |  |  |  |  |  |
| 12h | 0,76 | 0,75 | 0,74 | 0,69 | 0,69 | 0,70 | 0,66 | 0,75 | 0,83 | 0,88 | 0,94 | 0,96 | 1,00 |  |  |  |  |  |  |  |  |  |  |  |
| 13h | 0,70 | 0,68 | 0,68 | 0,63 | 0,63 | 0,65 | 0,61 | 0,69 | 0,77 | 0,83 | 0,90 | 0,92 | 0,96 | 1,00 |  |  |  |  |  |  |  |  |  |  |
| 14h | 0,69 | 0,67 | 0,67 | 0,62 | 0,62 | 0,64 | 0,61 | 0,66 | 0,73 | 0,79 | 0,86 | 0,89 | 0,93 | 0,95 | 1,00 |  |  |  |  |  |  |  |  |  |
| 15h | 0,61 | 0,60 | 0,59 | 0,57 | 0,58 | 0,61 | 0,57 | 0,59 | 0,63 | 0,69 | 0,75 | 0,77 | 0,82 | 0,84 | 0,92 | 1,00 |  |  |  |  |  |  |  |  |
| 16h | 0,40 | 0,40 | 0,40 | 0,39 | 0,41 | 0,41 | 0,39 | 0,37 | 0,40 | 0,44 | 0,51 | 0,50 | 0,57 | 0,60 | 0,71 | 0,84 | 1,00 |  |  |  |  |  |  |  |
| 17h | 0,40 | 0,41 | 0,41 | 0,41 | 0,43 | 0,42 | 0,40 | 0,41 | 0,42 | 0,44 | 0,51 | 0,50 | 0,55 | 0,59 | 0,67 | 0,77 | 0,91 | 1,00 |  |  |  |  |  |  |
| 18h | 0,44 | 0,45 | 0,45 | 0,45 | 0,46 | 0,46 | 0,42 | 0,46 | 0,45 | 0,47 | 0,52 | 0,54 | 0,59 | 0,62 | 0,69 | 0,76 | 0,82 | 0,90 | 1,00 |  |  |  |  |  |
| 19h | 0,51 | 0,51 | 0,50 | 0,53 | 0,53 | 0,52 | 0,49 | 0,52 | 0,49 | 0,50 | 0,55 | 0,56 | 0,60 | 0,61 | 0,68 | 0,78 | 0,74 | 0,80 | 0,89 | 1,00 |  |  |  |  |
| 20h | 0,53 | 0,53 | 0,52 | 0,53 | 0,53 | 0,52 | 0,50 | 0,54 | 0,53 | 0,54 | 0,58 | 0,60 | 0,62 | 0,63 | 0,69 | 0,75 | 0,70 | 0,76 | 0,83 | 0,91 | 1,00 |  |  |  |
| 21h | 0,91 | 0,91 | 0,89 | 0,85 | 0,83 | 0,80 | 0,75 | 0,74 | 0,74 | 0,77 | 0,77 | 0,74 | 0,74 | 0,69 | 0,69 | 0,62 | 0,44 | 0,41 | 0,46 | 0,51 | 0,52 | 1,00 |  |  |
| 22h | 0,94 | 0,94 | 0,92 | 0,89 | 0,87 | 0,85 | 0,82 | 0,80 | 0,79 | 0,80 | 0,79 | 0,75 | 0,74 | 0,70 | 0,68 | 0,61 | 0,43 | 0,42 | 0,47 | 0,51 | 0,53 | 0,97 | 1,00 |  |
| 23h | 0,97 | 0,96 | 0,94 | 0,92 | 0,89 | 0,87 | 0,85 | 0,83 | 0,82 | 0,83 | 0,83 | 0,77 | 0,75 | 0,70 | 0,68 | 0,59 | 0,41 | 0,42 | 0,46 | 0,49 | 0,51 | 0,92 | 0,96 | 1 |

Fonte de dados: INMET.

**Tabela S2: Comparação das especificações de modelos não lineares com temperatura máxima diária e mortalidade por todas as causas, utilizando o Critério de Informação de Akaike para modelos quasi-Poisson (Q-AIC).**

| **Knots (exposure–response)** | **Knots (lag–response)** | **Lag period** | **Q-AIC** |
| --- | --- | --- | --- |
| 50th and 90th | Three internal knots equally spaced | 0–21 days | 3.098.827 |
| 50th and 90th | Three internal knots log-spaced | 0–21 days | 3.099.217 |
| 50th, 75th, and 90th | Three internal knots equally spaced | 0–21 days | 3.103.590 |
| 10th, 50th, and 90th | Three internal knots equally spaced | 0–21 days | 3.103.687 |
| 25th, 50th, and 90th | Three internal knots equally spaced | 0–21 days | 3.103.784 |
| 10th, 75th, and 90th | Three internal knots equally spaced | 0–21 days | 3.103.945 |
| 10th, 50th, and 90th | Three internal knots log-spaced | 0–21 days | 3.103.981 |
| 25th, 50th, and 90th | Three internal knots log-spaced | 0–21 days | 3.104.008 |
| 50th, 75th, and 90th | Three internal knots log-spaced | 0–21 days | 3.104.113 |
| 10th, 75th, and 90th | Three internal knots log-spaced | 0–21 days | 3.104.143 |
| 50th and 90th | Three internal knots equally spaced | 0–14 days | 3.106.317 |
| 50th and 90th | Three internal knots log-spaced | 0–14 days | 3.106.569 |
| 50th and 90th | Three internal knots log-spaced | 0–10 days | 3.110.576 |
| 50th, 75th, and 90th | Three internal knots equally spaced | 0–14 days | 3.110.578 |
| 50th and 90th | Three internal knots equally spaced | 0–10 days | 3.110.612 |
| 50th, 75th, and 90th | Three internal knots log-spaced | 0–14 days | 3.111.034 |
| 10th, 50th, and 90th | Three internal knots equally spaced | 0–14 days | 3.111.129 |
| 10th, 50th, and 90th | Three internal knots log-spaced | 0–14 days | 3.111.337 |
| 25th, 50th, and 90th | Three internal knots equally spaced | 0–14 days | 3.111.398 |
| 25th, 50th, and 90th | Three internal knots log-spaced | 0–14 days | 3.111.459 |
| 10th, 75th, and 90th | Three internal knots equally spaced | 0–14 days | 3.111.522 |
| 10th, 75th, and 90th | Three internal knots log-spaced | 0–14 days | 3.111.531 |
| 50th and 90th | Three internal knots log-spaced | 0–7 days | 3.113.088 |
| 50th and 90th | Three internal knots equally spaced | 0–7 days | 3.113.297 |
| 50th, 75th, and 90th | Three internal knots log-spaced | 0–10 days | 3.114.689 |
| 50th, 75th, and 90th | Three internal knots equally spaced | 0–10 days | 3.114.716 |
| 10th, 50th, and 90th | Three internal knots log-spaced | 0–10 days | 3.115.346 |
| 25th, 50th, and 90th | Three internal knots log-spaced | 0–10 days | 3.115.411 |
| 10th, 50th, and 90th | Three internal knots equally spaced | 0–10 days | 3.115.503 |
| 50th and 90th | Three internal knots equally spaced | 0–5 days | 3.115.510 |
| 50th and 90th | Three internal knots log-spaced | 0–5 days | 3.115.510 |
| 10th, 75th, and 90th | Three internal knots log-spaced | 0–10 days | 3.115.572 |
| 25th, 50th, and 90th | Three internal knots equally spaced | 0–10 days | 3.115.598 |
| 10th, 75th, and 90th | Three internal knots equally spaced | 0–10 days | 3.115.748 |
| 50th, 75th, and 90th | Three internal knots equally spaced | 0–7 days | 3.117.717 |
| 50th, 75th, and 90th | Three internal knots log-spaced | 0–7 days | 3.117.929 |
| 10th, 50th, and 90th | Three internal knots log-spaced | 0–7 days | 3.118.075 |
| 10th, 75th, and 90th | Three internal knots log-spaced | 0–7 days | 3.118.077 |
| 25th, 50th, and 90th | Three internal knots log-spaced | 0–7 days | 3.118.178 |
| 10th, 50th, and 90th | Three internal knots equally spaced | 0–7 days | 3.118.202 |
| 10th, 75th, and 90th | Three internal knots equally spaced | 0–7 days | 3.118.255 |
| 25th, 50th, and 90th | Three internal knots equally spaced | 0–7 days | 3.118.301 |
| 50th, 75th, and 90th | Three internal knots equally spaced | 0–5 days | 3.120.271 |
| 50th, 75th, and 90th | Three internal knots log-spaced | 0–5 days | 3.120.271 |
| 10th, 50th, and 90th | Three internal knots equally spaced | 0–5 days | 3.120.352 |
| 10th, 50th, and 90th | Three internal knots log-spaced | 0–5 days | 3.120.352 |
| 25th, 50th, and 90th | Three internal knots equally spaced | 0–5 days | 3.120.417 |
| 25th, 50th, and 90th | Three internal knots log-spaced | 0–5 days | 3.120.417 |
| 10th, 75th, and 90th | Three internal knots equally spaced | 0–5 days | 3.120.465 |
| 10th, 75th, and 90th | Three internal knots log-spaced | 0–5 days | 3.120.465 |

**Tabela S3: Modelos de regressão linear entre temperaturas horárias e a Tmax durante episódios de ondas de calor em Belo Horizonte de 2014–2024**

| **Modelo** | **Preditores** | **R** | **R²** | **R² ajust.** | **Erro-padrão (°C)** | |
| --- | --- | --- | --- | --- | --- | --- |
| M1 | T06h | 0,46 | 0,21 | 0,20 | | 1,21 |
| M2 | T10h | 0,44 | 0,20 | 0,19 | | 1,22 |
| M3 | T14h | 0,80 | 0,64 | 0,64 | | 0,81 |
| M4 | T10h + T14h + T18h | 0,88 | 0,77 | 0,76 | | 0,66 |

Fonte de dados: INMET.

Figura S1: Índice de Calor do Serviço Nacional de Meteorologia. Fonte: NOAA (2025) - Adaptado. Nota: A conversão do limiar mínimo do Índice de Calor indica que 80 °F corresponde a aproximadamente 26,7 °C. Assim, quando a temperatura do ar é inferior a esse valor ou a umidade relativa é menor que 40%, o índice torna-se metodologicamente inválido.

Figura S2 - Anomalias das médias mensais (2014–2024) da Tmax e Tmin em relação às Normais Climatológicas 1991–2020 (A) e valores médios mensais absolutos (B). As linhas tracejadas indicam os percentis 25 e 75, e o sombreamento destaca o período de setembro a novembro, quando os desvios positivos de Tmax são maiores. Fonte de dados: INMET

**Figura S3 - Distribuição horária da temperatura máxima por níveis de alerta em Belo Horizonte, MG, de 2014 a 2024.** Fonte de dados: INMET.
