# Supplementary material for: Definition of mortality-associated thermal markers for an extreme heat alert system in Belo Horizonte, Minas Gerais, Brazil
Source: Rev Bras Epidemiol. 2026 Aug 28;29:e260048. doi: 10.1590/1980-549720260048 (PMC13524307; doi:10.1590/1980-549720260048)
Supplement: Translation

# Translation: Definição de marcadores térmicos associados à mortalidade para um sistema de alerta de calor extremo em Belo Horizonte, Minas Gerais, Brasil

**DOI:** 10.1590/1980-549720260048.2

**Authors:** Taíza Pinho Barroso Lucas, Amanda Silva Magalhães, Daniele Gomes Ferreira, Gabriel Andrade Camilo, Magda do Carmo Parajára, Fernanda Carvalho de Menezes, Aline Dayrell Ferreira Sales, Waleska Teixeira Caiaffa

**Affiliations:** I Prefeitura de Belo Horizonte, Coordenadoria Especial de Mudanças Climáticas – Belo Horizonte (MG), Brasil.; II Universidade Federal de Minas Gerais, Observatório de Saúde Urbana de Belo Horizonte – Belo Horizonte (MG), Brasil.; III Universidade Federal de Minas Gerais, Faculdade de Medicina, Programa de Pós-Graduação em Saúde Pública – Belo Horizonte (MG), Brasil.; IV Universidade Federal de Minas Gerais, Instituto de Geociências, Programa de Pós-Graduação em Geografia – Belo Horizonte (MG), Brasil.

**Keywords:** Ondas de calor, Temperatura ambiente, Séries temporais, Vigilância em saúde, Saúde urbana

## Abstract

**Objetivo:**

Estimar marcadores de temperatura máxima diária para definição de níveis de alerta de ondas de calor em Belo Horizonte, Minas Gerais, Brasil.

**Métodos:**

A associação entre temperatura máxima diária e mortalidade foi estimada por regressão quasi-Poisson com modelos de defasagem distribuída não linear, considerando defasagens de 0 a 21 dias e ajuste para tendência temporal, sazonalidade e dia da semana. A temperatura de mortalidade mínima foi utilizada como referência para estimativa de riscos relativos, e percentis elevados subsidiaram a definição de limiares operacionais.

**Resultados:**

A temperatura de mortalidade mínima foi 29,3°C. O risco de mortalidade aumentou nos percentis superiores, com riscos relativos de 1,04 no percentil 90; 1,09 no percentil 95; 1,17 no percentil 98; e 1,26 no percentil 99. Foram propostos quatro níveis baseados na temperatura máxima e na persistência térmica: Alerta 1, onda de calor leve (32,0–33,1°C/3 dias); Alerta 2, moderada (33,2–34,6°C/3 dias); Alerta 3, intensa (34,7–35,8°C/3 dias); e Alerta 4, extrema (>35,8°C/2 dias). Observou-se maior frequência de ondas de calor em anos associados ao El Niño.

**Conclusão:**

Marcadores baseados em percentis elevados da temperatura máxima produziram níveis coerentes com o aumento do risco de mortalidade, com potencial aplicação na vigilância e resposta em saúde pública.

## INTRODUÇÃO

As ondas de calor têm apresentado aumento em frequência e intensidade no contexto das mudanças climáticas, configurando-se como um dos extremos climáticos de maior impacto sobre a saúde pública^1,2^. No ambiente urbano brasileiro, sua ocorrência extrapola a dimensão meteorológica, produzindo impactos diferenciados em populações expostas a desigualdades de moradia, infraestrutura urbana e acesso aos serviços de saúde^3,4^.

Eventos históricos em diferentes países evidenciaram aumento expressivo da morbimortalidade associada à exposição prolongada ao calor extremo, como o episódio de Chicago em 1995^5,6^ e a onda de calor europeia de 2003, responsável por dezenas de milhares de óbitos adicionais^7^. Evidências epidemiológicas e revisões sistemáticas indicam associação consistente entre temperaturas elevadas e aumento da mortalidade, sobretudo por causas cardiovasculares e respiratórias, além de morbidades como agravos renais, desidratação, alterações hidroeletrolíticas, internações e atendimentos de urgência^8–12^. Esses impactos tendem a ser mais intensos entre idosos, crianças, trabalhadores expostos ao calor e populações socialmente vulneráveis^13–16^.

Não há definição universal de "onda de calor", uma vez que sua caracterização depende da climatologia local e dos impactos associados. Diretrizes internacionais e estudos em saúde pública recomendam abordagens orientadas ao risco, baseadas em limiares térmicos relativos à distribuição histórica local^2,17^. No Brasil, o critério operacional adotado pelo Instituto Nacional de Meteorologia (Inmet) define onda de calor como um período de cinco dias ou mais em que as temperaturas máximas diárias excedem em pelo menos 5°C a média climatológica local^18^. Embora amplamente utilizada, essa abordagem pode ser limitada para fins epidemiológicos, ao passo que estudos baseados em percentis térmicos têm demonstrado maior adequação para incorporar especificidades climáticas locais, caracterizar ondas de calor em diferentes contextos e avaliar seus impactos sobre a saúde^19–22^.

A persistência do calor, a baixa umidade relativa, a poluição atmosférica e condições adversas podem potencializar riscos à saúde durante eventos extremos^2,22^. Assim, estudos destacam que a definição de ondas de calor para fins epidemiológicos e operacionais deve considerar características climáticas locais e sua associação com desfechos em saúde, pois não existe limiar térmico universal aplicável a diferentes regiões^23^. Abordagens baseadas em percentis elevados e indicadores de risco relativo têm sido amplamente utilizadas na formulação de sistemas de alerta e protocolos de resposta em saúde pública^17,24,25^.

No Brasil, Bitencourt et al. apontaram aumento da frequência, duração e intensidade das ondas de calor nas últimas décadas, associado tanto à variabilidade climática quanto ao aquecimento regional^21^. Belo Horizonte (BH), uma das maiores capitais brasileiras, reúne condições que dificultam a gestão ambiental, como alta densidade urbana, verticalização em áreas centrais e desigualdade na distribuição da cobertura vegetal^26^. A cidade tem registrado aumento de eventos extremos de calor, sobretudo na primavera, período de transição para a estação úmida, característico do clima Tropical Continental^27^. A variabilidade climática associada ao El Niño-Oscilação Sul (ENOS) também pode influenciar os extremos térmicos e pluviométricos no Brasil, com intensificação das anomalias de temperatura durante sua fase quente^28^.

Diversos países estruturaram sistemas de alerta ajustados às suas realidades climáticas e orientados à prevenção de impactos à saúde^17,24^. No Reino Unido, o sistema evoluiu para uma orientação explícita ao impacto à saúde^29^, enquanto Hong Kong desenvolveu o Hong Kong Heat Index^30^. No Brasil, o Rio de Janeiro adotou um índice que combina temperatura e umidade relativa para orientar ações preventivas^31^.

Em BH, o aprimoramento do Plano Municipal de Gestão de Riscos e Desastres Climáticos demanda parâmetros epidemiologicamente fundamentados para orientar respostas intersetoriais ao calor extremo. Este estudo teve como objetivo estabelecer limiares de temperatura máxima diária para definição de níveis de alerta e caracterização de ondas de calor em Belo Horizonte, fornecendo base teórico-metodológica para a gestão de riscos climáticos no município.

## MÉTODOS

### Área de estudo

BH localiza-se no Sudeste do Brasil, tem 331,4 km^2^ e abriga aproximadamente 2,3 milhões de habitantes^32^. O clima enquadra-se no Tropical Continental de Altitude, inserido no Subdomínio Tropical Montano e no Domínio Tropical Brasil Central, conforme classificação atualizada da climatologia brasileira proposta por Novais^27^.

As características físicas da cidade se distinguem entre as porções sudoeste-sul e norte. A porção sul apresenta formações do Supergrupo Minas, compostas por rochas metassedimentares resistentes ao intemperismo, associadas a relevo ondulado e dissecado, com altitudes superiores a 1.300 m^33^. Essas variações geomorfológicas modulam o comportamento térmico urbano, sobretudo em áreas adjacentes à Serra do Curral, que apresentam menores temperaturas médias e maior volume de precipitação em comparação com outras áreas do município^34^. A interação entre topografia, altitude, gradientes térmicos e a baixa umidade relativa associada aos períodos de atuação do Anticiclone Subtropical do Atlântico Sul (ASAS), somada à elevada impermeabilização do solo, contribui para padrões distintos de exposição ao calor no território municipal^35^.

### Fontes de dados

Os dados de mortalidade foram obtidos do Sistema de Informação sobre Mortalidade (SIM). Foram extraídos os registros diários de óbitos por todas as causas de residentes de BH, abrangendo os anos de 2014–2024. A opção pela mortalidade geral visa capturar o impacto sistêmico da exposição ao calor sobre a saúde pública municipal.

Os dados meteorológicos são provenientes da rede de monitoramento do Inmet. O município dispõe de uma estação convencional e de duas automáticas, instaladas em setores com características geoecológicas distintas. Selecionou-se a Estação Automática da Pampulha (EAP; Código A521; lat: −19,88; long: −43,97), na porção noroeste da capital, pela representatividade espacial e altimétrica, a 854 m de altitude, cota próxima ao valor médio do sítio urbano belo-horizontino. Apesar da morfologia urbana complexa e da formação de microclimas, a EAP ocupa posição estratégica em relação ao centroide da mancha urbana, sintetizando os parâmetros climáticos da cidade. Foram utilizados os registros diários de temperatura máxima (Tmax) e mínima (Tmin), bem como as respectivas médias mensais, para 2014–2024. As anomalias térmicas foram calculadas com base nas Normais Climatológicas de 1991-2020^36^.

### Análises estatísticas

#### Etapa 1

Realizou-se inicialmente uma análise exploratória para caracterizar o comportamento das Tmax e Tmin no período de 2014–2024, contrastando-as com as Normais Climatológicas de 1991–2020^36^. Também foram calculados os percentis 25 e 75 das temperaturas máxima e mínima mensais, para descrever a variabilidade térmica do período analisado. Identificaram-se episódios de ondas de calor com base no desvio térmico de 5°C acima da média climatológica de referência, conforme critério adotado pelo Inmet^18^. Neste estudo, a persistência temporal foi ajustada para três dias consecutivos.

A utilização de percentis elevados da distribuição da Tmax complementou a abordagem climatológica do Inmet, incorporando especificidades locais e permitindo avaliar sua associação com o risco relativo de mortalidade no município. Estudos demonstram que abordagens baseadas em percentis térmicos apresentam maior adequação para análises epidemiológicas e definição de limiares locais de risco^19–23^.

Essa calibração fundamenta-se nas diretrizes da Organização Mundial da Saúde (OMS) e da Organização Meteorológica Mundial (OMM), que recomendam adaptar os critérios de definição de ondas de calor às especificidades climáticas locais e aos impactos sobre a saúde^17^. Ressalta-se que o limiar de três dias consecutivos já é adotado institucionalmente pela Defesa Civil Municipal na gestão de riscos de Belo Horizonte, conferindo aplicabilidade operacional aos marcadores propostos.

Após a identificação dos episódios, verificou-se a influência da variabilidade climática interanual, especificamente das fases do El Niño e da La Niña^28,37^. A classificação dos eventos ENOS baseou-se no *Oceanic Niño Index* (ONI), calculado com base nas anomalias trimestrais da temperatura da superfície do mar na região Niño 3.4 do Pacífico Equatorial, conforme critérios da National Oceanic and Atmospheric Administration (NOAA)^37^. Eventos classificados como muito fortes corresponderam a valores de ONI ≥2,0°C.

Para as modelagens subsequentes, optou-se pelo uso da Tmax, dada sua ampla utilização na caracterização de extremos de calor^19–22^ e sua relação com o aumento da mortalidade descrita em estudos epidemiológicos^8–12^. Essa escolha permitiu preservar a variabilidade temporal diária necessária à estimativa dos efeitos associados ao calor extremo.

#### Etapa 2 – Identificação de marcadores de calor persistente

Para definir o marcador térmico mais representativo dos eventos extremos, realizaram-se análises de correlação e regressão linear baseadas nos episódios identificados na Etapa 1. Aplicou-se inicialmente a correlação de Pearson entre a Tmax e as temperaturas de bulbo seco observadas às 6h, 10h, 14h e 18h, horários representativos do amanhecer, do aquecimento diurno, do pico térmico da tarde e do início do resfriamento noturno. Posteriormente, ajustaram-se modelos de regressão linear simples e múltipla para avaliar o poder explicativo de combinações horárias do ciclo diário na predição da Tmax. As correlações horárias completas são apresentadas na Tabela S1 (material suplementar).

#### Etapa 3 – Variação intra e interdiária da temperatura

Investigou-se a variação da Tmax ao longo do ciclo diurno e entre dias por meio de um Modelo Aditivo Generalizado (GAM)^38^, com finalidade descritiva. A Tmax foi definida como variável resposta, e a hora do dia e a sequência temporal, como covariáveis. O modelo utilizou *splines* cíclicos para a hora do dia e *splines* cúbicos penalizados para a dimensão temporal, permitindo ajustar tendências sazonais e temporais. Essa abordagem permitiu modelar variações intra e interdiárias sem superajuste, caracterizando a persistência térmica durante os episódios de ondas de calor^38^.

#### Etapa 4 – Modelagem da associação entre temperatura e mortalidade

Empregou-se o modelo não linear de defasagem distribuída (*Distributed Lag Non-linear Model* – DLNM), com distribuição quasi-Poisson^39,40^, para estimar a associação entre temperatura do ar e mortalidade por todas as causas. Na dimensão exposição-resposta, utilizou-se *spline* cúbico natural com nós internos nos percentis 50 e 90 da distribuição térmica^39,40^. Para a dimensão defasagem-resposta, aplicou-se *spline* cúbico natural com três nós internos igualmente espaçados na escala logarítmica, considerando defasagem de 0 a 21 dias^39,41^. O ajuste para sazonalidade e tendências de longo prazo foi feito por uma variável de estrato temporal composta por ano, mês e dia da semana. A seleção das especificações finais baseou-se no menor valor do Critério de Informação de Akaike (Q-AIC), conforme detalhado na Tabela S2 (material suplementar).

Previamente à escolha da Tmax como preditor principal, avaliou-se a aplicabilidade do índice *Heat Adjusted Air Temperature* (HAAT). Para essa avaliação, analisaram-se as correlações entre a Tmax diária, as temperaturas de bulbo seco às 6h, 10h, 14h e 18h durante os episódios identificados na Etapa 1 e os respectivos valores de umidade relativa do ar. Contudo, a análise demonstrou que a conversão do limiar mínimo da NOAA (80°F, aproximadamente 26,7°C) e a exigência de Umidade Relativa (UR) ≥40% inviabilizam a aplicação do índice ao regime climático de Belo Horizonte, marcado por baixa umidade, especialmente durante as ondas de calor (Figura S1 – material suplementar)^42^. Essa limitação difere das condições observadas em estudos de calor e mortalidade no Rio de Janeiro^43^, onde a maritimidade exerce maior influência sobre os parâmetros térmicos locais.

#### Etapa 5 – Limiares de risco e parâmetros para o Protocolo de Calor

Com base nos resultados do modelo DLNM, estimou-se a Temperatura de Mortalidade Mínima (TMM), definida como o valor associado ao menor risco de mortalidade. Para evitar estimativas instáveis nos extremos da distribuição, a TMM foi limitada aos percentis 1 a 99 da Tmax diária^44^. Os Riscos Relativos (RR) e os Intervalos de Confiança de 95% (IC 95%) foram estimados usando a TMM como referência.

Para avaliar a robustez dos achados, realizaram-se análises de sensibilidade testando distintas posições de nós na dimensão exposição-resposta e diferentes períodos de defasagem, priorizando-se o modelo com melhor ajuste global via Q-AIC^45^. Com base nos RR estimados, na TMM e na persistência de dias consecutivos de calor extremo, definiram-se os parâmetros térmicos que subsidiaram os níveis de alerta do Protocolo de Calor do município. As análises foram realizadas no R (versão 4.4.2), com os pacotes dlnm^39^ e gnm^46^, e o processamento descritivo complementar em planilhas eletrônicas.

##### Declaração de disponibilidade de dados:

Todo o conjunto de dados que dá suporte aos resultados deste estudo está disponível mediante solicitação ao autor correspondente. O conjunto de dados não está publicamente disponível devido à integração de bancos de dados realizados pelos autores.

## RESULTADOS

### Etapa 1

Os dados meteorológicos de 2014–2024 evidenciaram desvios sistemáticos em relação às Normais Climatológicas de 1991–2020. A temperatura máxima média do período foi de 28,4°C, cerca de 1°C acima do padrão histórico, enquanto a média das mínimas (17,6°C) manteve-se ligeiramente abaixo da climatológica.

As anomalias mensais da Tmax revelaram predomínio de desvios positivos ao longo dos anos analisados (Figura S2A – material suplementar), variando de +0,2°C a +2,3°C. Esse aquecimento foi mais pronunciado no trimestre setembro-novembro, com pico médio em setembro (aproximadamente +2,3°C), caracterizando a intensificação e o prolongamento do calor na transição para o período chuvoso. Tal comportamento foi confirmado pelos valores absolutos (Figura S2B), nos quais as médias das máximas superaram consistentemente 30°C nesse período, evidenciando elevada exposição sazonal ao calor da população de Belo Horizonte.

Em contraste, a Tmin apresentou anomalias negativas persistentes entre março e agosto (Figura S2A), com mínimo médio em julho (-1,3 a −1,5°C), indicando maior resfriamento noturno nos meses secos. A partir de setembro, observou-se inflexão no padrão térmico, com elevação gradual da Tmin até dezembro, aproximando-se ou superando as normais climatológicas na maioria dos anos, exceto em 2021 e 2022. Esse padrão evidencia aquecimento assimétrico, dominado pelo incremento das temperaturas máximas, especialmente na transição inverno-primavera (Figura S2B). Tal assimetria caracteriza a exposição térmica urbana, ao evidenciar maior contribuição das temperaturas máximas diurnas nos episódios de calor extremo.

Com base nos registros diários de Tmax, identificaram-se as ondas de calor anuais (Tabela 1). O ano de 2015, sob influência de El Niño muito forte segundo o índice ONI, registrou o maior número de eventos da série, concentrados entre setembro e novembro. Os anos de 2019 e 2024 também apresentaram elevada recorrência de ondas de calor, associada à atuação de fases quentes do ENOS. Em contraste, anos sob influência de La Niña apresentaram menor persistência térmica e menor frequência de episódios classificados nos níveis superiores de alerta. Embora 2023 tenha contabilizado apenas três eventos, concentrou a onda de calor de maior duração e intensidade do período analisado. Nesse período, registrou-se a Tmax absoluta da série (38,4°C), excedendo em mais de 10°C a normal climatológica mensal, acompanhada de elevações concomitantes nas temperaturas mínimas.

**Tabela 1:** Ondas de calor em Belo Horizonte (MG), de 2014 a 2024.

Ano	Data inicial	Data final	Número de dias
2014	29/09/2014	01/10/2014	3
	12/10/2014	15/10/2014	4
	17/10/2014	19/10/2014	3*
2015	22/09/2015	27/09/2015	6*
	14/10/2015	17/10/2015	4*
	20/10/2015	22/10/2015	3*
	03/11/2015	07/11/2015	5*
	10/11/2015	13/11/2015	4*
2016	19/10/2016	21/10/2016	3†
2017	20/10/2017	22/10/2017	3†
2018	-	-	-
2019	10/09/2019	13/09/2019	4
	17/09/2019	21/09/2019	5
	04/11/2019	06/11/2019	3
	08/11/2019	11/11/2019	4
2020	18/09/2020	20/09/2020	3†
	27/09/2020	03/10/2020	7†
	06/10/2020	09/10/2020	4†
2021	09/09/2021	11/09/2021	3†
	13/09/2021	15/09/2021	3†
2022	-	-	-
2023	11/07/2023	13/07/2023	3*
	23/09/2023	28/09/2023	6*
	09/11/2023	19/11/2023	11*
2024	01/05/2024	03/05/2024	3*
	12/05/2024	14/05/2024	3*
	23/08/2024	25/08/2024	3
	02/09/2024	05/09/2024	4

* Ocorrência de El Niño;

† La Niña.

Fonte: Instituto Nacional de Meteorologia e National Oceanic and Atmospheric Administration.

### Etapa 2

A Tabela S3 (material suplementar) apresenta os modelos de regressão que relacionam as temperaturas horárias à Tmax durante episódios de ondas de calor (2014–2024). A temperatura das 6h (T06h) apresentou correlação moderada com a Tmax (R = 0,46; R² = 0,21), com desempenho semelhante ao da temperatura das 10h (R = 0,44; R² = 0,20). Em contraste, a temperatura das 14h (T14h) demonstrou forte associação com a Tmax (R = 0,80; R² = 0,64) e apresentou o menor erro-padrão individual (0,81°C). O modelo múltiplo (M4), que integrou as temperaturas das 10h, 14h e 18h, apresentou o melhor ajuste (R² ajustado = 0,76), indicando que a persistência térmica ao longo do ciclo diurno é um determinante-chave da magnitude das ondas de calor no município.

### Etapa 3

A modelagem via GAM confirmou um padrão diurno consistente durante os episódios de calor extremo. O comportamento observado corrobora os achados da Etapa 2, consolidando a temperatura das 14h como marcador central da Tmax em Belo Horizonte. Verificou-se elevação térmica progressiva no período matutino, com platô máximo entre 14h e 16h, seguido de declínio gradual no período noturno. O ajuste do *spline* temporal evidenciou persistência térmica entre dias sucessivos, reforçando a natureza prolongada da exposição ao calor durante os episódios de calor extremo.

### Etapa 4

No período analisado, a média diária de óbitos por todas as causas foi de 47,6. O incremento de mortalidade observado em 2020 associou-se ao contexto pandêmico, sem correlação com extremos térmicos. Em contraste, os episódios de calor de setembro de 2015 e, mais notadamente, de novembro de 2023 exibiram padrão consistente de elevação da mortalidade concomitante aos picos de Tmax e Tmin e à queda acentuada da UR (Figura 1). Durante as ondas de calor, todos os valores de UR ficaram abaixo de 40%.

**Figura 1:** Eventos de ondas de calor em Belo Horizonte (MG) (2014-2024) e relação com mortalidade e variáveis meteorológicas.

Colunas: número máximo diário de óbitos por evento; área sombreada: média de óbitos por período; linhas: temperatura máxima (Tmax) e mínima (Tmin); barras azuis: umidade relativa mínima; eixo inferior: duração (n^o^ de dias).

Fonte: Instituto Nacional de Meteorologia e Sistema de Informação sobre Mortalidade/DataSUS.

A análise estratificada revelou que aproximadamente 40% dos dias avaliados apresentaram temperaturas acima da temperatura de mortalidade mínima (TMM=29,3°C). Observou-se efeito dose-resposta progressivo: a média de óbitos elevou-se de 48,0/dia (acima da TMM) para 55,1/dia nos dias com Tmax ≥35,8°C (P99), conforme a Tabela 2.

**Tabela 2:** Número de dias e média diária de óbitos por faixa de temperatura (2014–2024) em Belo Horizonte (MG), Brasil.

Tmax (°C)	Número de dias	Média de mortes por dia
≥29,3°C (TMM; P59)	1626	48,0
≥32,0°C (P90)	423	48,7
≥33,1°C (P95)	197	49,8
≥34,6°C (P98)	80	51,4
≥35,8°C (P99)	40	55,1

Fonte: Instituto Nacional de Meteorologia e Sistema de Informação sobre Mortalidade/DataSUS.

A modelagem via DLNM confirmou a relação não linear e o formato em "U" da curva de risco (Figura 2). Acima da TMM (P59), o RR de mortalidade apresentou crescimento acentuado em percentis elevados: RR 1,09 (IC95% 1,04–1,14) no P95, atingindo RR 1,26 (IC95% 1,13–1,41) no P99, considerando o efeito cumulativo de 0 a 21 dias.

**Figura 2:** Associação cumulativa entre a Tmax e a mortalidade por todas as causas em Belo Horizonte (MG) (*lag* 0-21 dias), tendo como referência a Temperatura de Mortalidade Mínima.

A linha contínua representa a curva exposição-resposta e a área sombreada, o intervalo de confiança de 95%. A linha tracejada azul indica a Temperatura de Mortalidade Mínima (percentil 59); as linhas pontilhadas verdes, os nós do modelo (percentis 50 e 90); e as linhas tracejadas-ponto vermelhas, os percentis extremos (95, 98 e 99).

Fonte: Instituto Nacional de Meteorologia e Sistema de Informação sobre Mortalidade/DataSUS.

### Etapa 5

Após a modelagem DLNM, definiram-se os níveis de alerta para o Protocolo de Belo Horizonte (Tabela 3), adotando a Tmax como variável de referência. Os limiares térmicos basearam-se na TMM (29,3°C) e nos percentis 90, 95, 98 e 99, que apresentaram aumento progressivo do risco relativo de mortalidade.

**Tabela 3:** Níveis de alerta de calor em Belo Horizonte (MG), Brasil.

Nível/Alerta	Critérios para o nível de alerta (Tmax e n^o^ de dias consecutivos)	Classificação	Risco à saúde
Inicial	29,3°C<Tmax≤31,9°C	ATENÇÃO	Risco muito baixo
Alerta 1	32,0°C≤Tmax≤33,1°C 3 dias consecutivos	ONDA DE CALOR LEVE	Risco baixo
Alerta 2	33,2°C≤Tmax≤34,6°C 3 dias consecutivos	ONDA DE CALOR MODERADA	Risco moderado
Alerta 3	34,7°C≤Tmax≤35,8°C 3 dias consecutivos	ONDA DE CALOR INTENSA	Risco alto
Alerta 4	Tmax>35,8°C 2 dias consecutivos	ONDA DE CALOR EXTREMA	Risco muito alto

Fonte: Instituto Nacional de Meteorologia e Sistema de Informação sobre Mortalidade/DataSUS.

O nível inicial (Atenção) compreende o intervalo imediatamente superior à TMM (29,3 a 31,9°C). Para os níveis de Alerta 1, 2 e 3 (ondas de calor leve, moderada e intensa), estabeleceu-se o critério de três dias consecutivos com temperaturas entre 32,0–33,1°C, 33,2–34,6°C e 34,7–35,8°C, respectivamente. Para o nível de Alerta 4 (extremo), adotou-se o limiar de dois dias consecutivos acima de 35,8°C (P99), em razão do risco crítico observado nesse percentil.

A distribuição horária confirma padrão diurno bem definido, com concentração dos picos térmicos entre 12h e 17h e valor máximo entre 14h e 15h (Figura S3 – material suplementar). Esse padrão é particularmente evidente no nível de Alerta 4, reforçando a Tmax como o marcador preditivo mais robusto para a operacionalização do sistema de alerta e definição de janelas de maior vulnerabilidade térmica em BH.

## DISCUSSÃO

Os resultados sugerem associação entre a elevação da Tmax, a persistência de dias consecutivos de calor extremo e o aumento da mortalidade em Belo Horizonte, sem pressupor uniformidade entre os episódios analisados. Esse padrão reforça as limitações de definições exclusivamente meteorológicas de ondas de calor quando aplicadas à saúde pública e sustenta a adoção de critérios baseados em impacto, calibrados às especificidades climáticas locais, conforme preconizado pela OMS e pela OMM^17,23^.

A relação não linear entre temperatura e mortalidade, com formato em "U" e TMM em 29,3°C, converge com evidências de estudos multicêntricos sobre temperatura e mortalidade^40,44^. Em Belo Horizonte, o aumento do risco foi mais acentuado em temperaturas acima da TMM, especialmente nos percentis superiores da Tmax. O incremento progressivo do risco relativo a partir do P90, atingindo RR 1,26 no P99 (>35,8°C), fornece base empírica para a diferenciação dos níveis de alerta e para a incorporação da persistência térmica como critério operacional^22,23,45^.

A inaplicabilidade do índice HAAT em Belo Horizonte decorre da elevada variabilidade climática local e da persistência de baixa umidade relativa durante os episódios de calor extremo. Essa condição difere da observada no Rio de Janeiro, onde a maritimidade exerce maior influência sobre os parâmetros térmicos e favorece o uso de índice que combina temperatura e UR^31,43^. Em Belo Horizonte, os episódios de calor extremo associam-se frequentemente à estabilidade atmosférica, à subsidência, à redução da nebulosidade e à baixa umidade relativa do ar no Sudeste brasileiro^47^. Esse padrão reforça a Tmax como marcador para o sistema de alerta local.

Como cidade de clima Tropical Continental de Altitude, Belo Horizonte apresenta forte sazonalidade, com picos de calor na primavera, período de transição para o regime úmido^27^. A análise horária da Figura S3 (material suplementar) reforça esse comportamento ao indicar concentração dos valores máximos entre 12h e 17h, período de maior exposição populacional em atividades laborais e deslocamentos urbanos. A maior recorrência de ondas de calor em anos associados ao El Niño é compatível com evidências sobre a influência das fases quentes do ENOS nos extremos térmicos e pluviométricos no Brasil^28^. A seca severa de 2014–2015 no Sudeste brasileiro e o aquecimento excepcional observado em 2023 reforçam a relevância da variabilidade climática interanual na intensificação de extremos térmicos no Brasil, aspecto a ser considerado no aprimoramento de sistemas de alerta e resposta em saúde pública^17,47–49^.

Os resultados dialogam com experiências internacionais e nacionais de sistemas de alerta calor-saúde, incluindo o Rio de Janeiro, que também adota níveis graduados de risco^17,24,29,31^. A proposta para Belo Horizonte diferencia-se ao ancorar os limiares na Tmax e na persistência térmica, refletindo particularidades da continentalidade, altitude e sazonalidade local. Essa abordagem favorece a comunicação institucional e a tomada de decisão em cenários de variabilidade climática, como episódios de El Niño, e em previsões semanais. Reconhece-se como limitação o uso de uma única estação meteorológica, restringindo a captação da heterogeneidade térmica intraurbana decorrente do relevo, da cobertura vegetal e da ocupação do solo^26,33,34^. Pesquisas futuras com dados microclimáticos e análises estratificadas por grupos vulneráveis poderão refinar espacialmente os limiares propostos. Ainda assim, os marcadores definidos oferecem uma régua operacional para vigilância e resposta em saúde pública, contribuindo para atuação institucional mais preventiva diante dos eventos de calor extremo.

## Material suplementar

Material suplementar
